# Rapid Detection of Pomelo Fruit Quality Using Near-Infrared Hyperspectral Imaging Combined With Chemometric Methods

**DOI:** 10.3389/fbioe.2020.616943

**Published:** 2021-01-12

**Authors:** Huazhou Chen, Hanli Qiao, Quanxi Feng, Lili Xu, Qinyong Lin, Ken Cai

**Affiliations:** ^1^College of Science, Guilin University of Technology, Guilin, China; ^2^Center for Data Analysis and Algorithm Technology, Guilin University of Technology, Guilin, China; ^3^College of Marine Sciences, Beibu Gulf University, Qinzhou, China; ^4^College of Automation, Zhongkai University of Agriculture and Engineering, Guangzhou, China

**Keywords:** near-infrared hyperspectral imaging (NIRHI), pomelo fruit quality, agricultural product, chemometric method, partial least squares (PLS), Gaussian radial basis function (RBF)

## Abstract

Pomelo is an important agricultural product in southern China. Near-infrared hyperspectral imaging (NIRHI) technology is applied to the rapid detection of pomelo fruit quality. Advanced chemometric methods have been investigated for the optimization of the NIRHI spectral calibration model. The partial least squares (PLS) method is improved for non-linear regression by combining it with the kernel Gaussian radial basis function (RBF). In this study, the core parameters of the PLS latent variables and the RBF kernel width were designed for grid search selection to observe the minimum prediction error and a relatively high correlation coefficient. A deep learning architecture was proposed for the parametric scaling optimization of the RBF-PLS modeling process for NIRHI data in the spectral dimension. The RBF-PLS models were established for the quantitative prediction of the sugar (SU), vitamin C (VC), and organic acid (OA) contents in pomelo samples. Experimental results showed that the proposed RBF-PLS method performed well in the parameter deep search progress for the prediction of the target contents. The predictive errors for model training were 1.076% for SU, 41.381 mg/kg for VC, and 1.136 g/kg for OA, which were under 15% of their reference chemical measurements. The corresponding model testing results were acceptably good. Therefore, the NIRHI technology combined with the study of chemometric methods is applicable for the rapid quantitative detection of pomelo fruit quality, and the proposed algorithmic framework may be promoted for the detection of other agricultural products.

## Introduction

Pomelo is one of the special agricultural fruit products that is popular in southern China. Its scientific name is *Citrus maxima (Brum.) Merr*. Ripe pomelo fruits are picked, stored, and served for eating. The fruit peel has functional curative effects in traditional Chinese medicine (Jiang et al., 2014). The flesh is edible and tastes delicious, sweet, and slightly sour; it is rich in sugar, vitamin C, and organic acids, which provide a variety of nutrients for the human body (Sirisomboon and Lapcharoensuk, 2012). People's health can be partially improved from the consumption of good-quality pomelo fruits. Eating pomelo can help maintain good stomach digestion ability and exerts an auxiliary effect of preventing influenza (Anlamlert et al., 2015). Thus, the pomelo fruit quality should be determined during the picking and storage process. Conventional laboratory methods for detecting chemical contents are tedious and time consuming. Rapid detection technology is appreciably on demand (Xu et al., 2020).

Hyperspectral imaging (HI) is regarded as an emerging advanced analytical technology for the non-destructive rapid determination of agricultural product quality (ElMasry et al., 2012; Barreto et al., 2018). HI generates two-dimensional spatial digital imagery accompanied with spectroscopic records for the analysis of spectral features in the ultraviolet, visible, near-infrared, or infrared regions. It technically supports signal processing in the field of computer vision (Lorente et al., 2012). The near-infrared (NIR) spectral region (around 800–2,500 nm) provides a versatile range of light frequencies to analyze the molecule structure and quantify their substantial contents (Pojić and Mastilović, 2013). The recognition of informative features from the natural overlapping signals requires the investigation of smart chemometric methods in the modern intelligent world (Sciutto et al., 2014; Cheng and Sun, 2015). On this basis, HI technology originating from the NIR region (denoted as NIRHI for short) facilitates the combination analysis of the imaging pixels and the NIR-range spectral data (Costa et al., 2011; Cheng et al., 2014). This technology is used as an advanced tool for qualitative and quantitative analyses in the fields of agriculture, food, and industry (Wu and Sun, 2013; Verdú et al., 2016; Arendse et al., 2018). Research on the quality detection of bakery food, meat, and fresh vegetables (Kamruzzaman et al., 2016; Erkinbaev et al., 2017) has been published, but quality assessments for fruits are a brand-new emerging application (Munera et al., 2017).

In NIRHI analysis, the spatial pixels include rich spectral information, and the spectral signals can be used for the rapid quantitative determination of any nutrient content in agricultural products. The selection of the spatial region of interest (ROI) and studies on chemometric methods to extract informative latent variables in the spectral dimension are both significant for NIRHI technology. Given that the selection of ROI has been studied extensively (Chen et al., 2019), feature extraction in the spectral dimension is the main focus of this study.

For the analysis of spectral data, partial least square (PLS) regression is a classical method in finding the latent variables that reflect most of the information of the target analytes. PLS performs principle component extraction, followed by linear regression on the component variables (Wold et al., 2001; Jin and Wang, 2019). However, for NIRHI analysis of complex objects such as pomelo fruit, the spectral dimension contains signal responses from all chemical compositions. The regression model does not stand as a linear formula for a few target analytes. A non-linear kernel function should then be introduced as an algorithmic embedment of PLS (Kim et al., 2005). The Gaussian radial basis function (RBF) is most commonly used for mapping data into a higher dimensional data space for linear fitting (Sandberg, 2003). Its effectiveness and fast tuning of the kernel width ensure that RBF is a successful algorithm application in the kernel PLS method (Shariati-Rad and Hasani, 2013; de Almeida et al., 2018).

In this work, NIRHI technology was applied for the rapid detection of the pomelo fruit quality during its picking and storage processes. The RBF-implemented PLS (RBF-PLS) method was investigated as an advanced chemometric method for the quantitative determination of the sugar (SU), vitamin C (VC), and organic acid (OA) contents. A deep learning architecture was built for parametric scaling optimizations. The model training procedure was launched by automatically tuning the PLS parameters in combination with the machine learning of the RBF kernel width, and the optimal model was tested based on the assumed pseudo-unknown samples. In this way, NIRHI may be considered a modern popular technology for detecting the fruit quality of agricultural products.

## Materials and Methods

### Hyperspectral System

**Figure 1:**
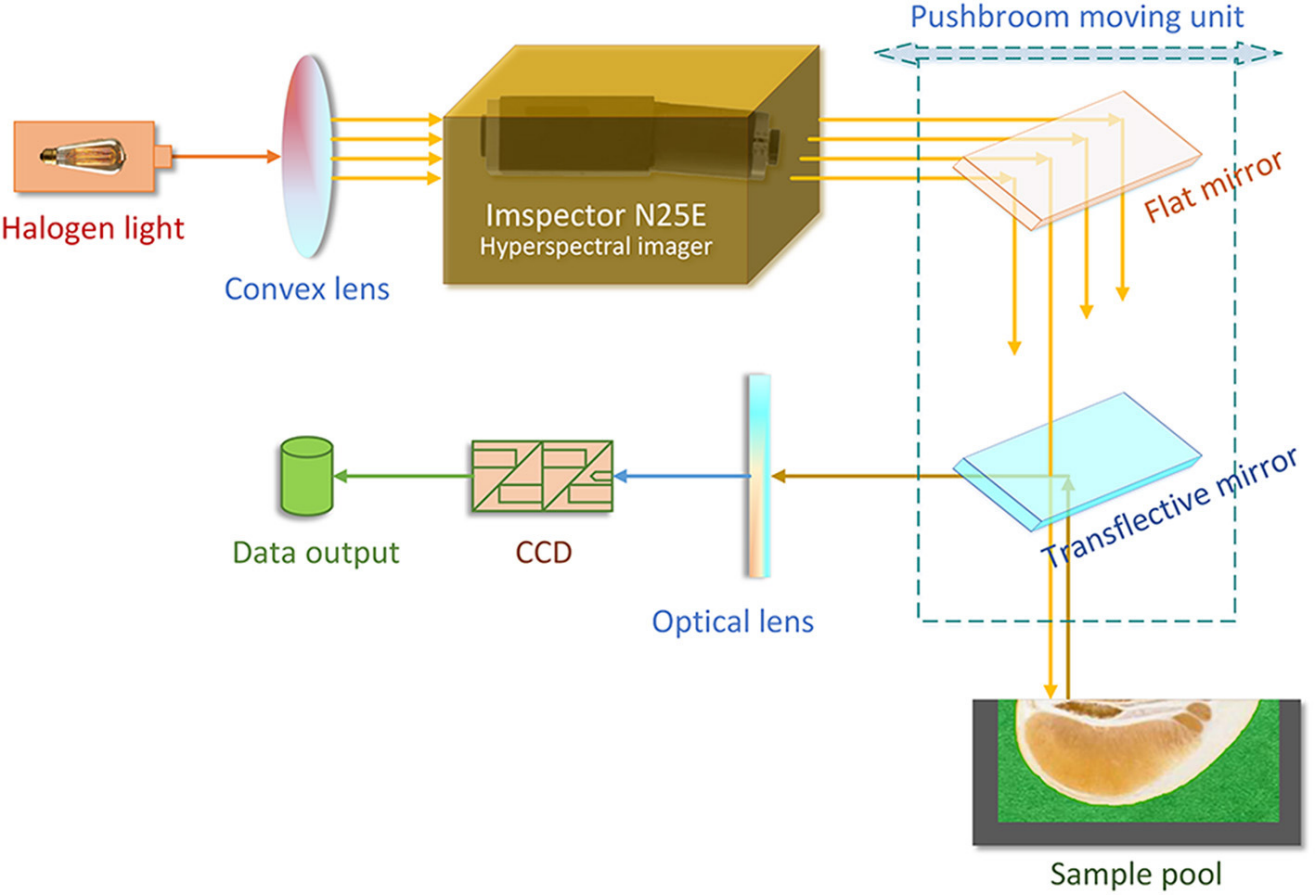
The construction of the NIRHI optical system.

As shown in Figure 1, the NIRHI optical system was constructed under laboratory conditions with constant temperature and humidity (25 ± 1°C and 47 ± 1%RH). The NIR lights were originally generated from a 500 W-powered halogen light source. The halogen light was transformed into a series of parallel lights via a convex lens (with 30 mm focal length). The ImSpector N25E hyperspectral imager (Spectral Imaging Ltd., Oulu, Finland) is the main optical part, which splits the source halogen light into a single frequency and produces a batch of NIR wavelengths according to the system pre-settings. The N25E imager generates the full-length NIR waveband of 1,000–2,500 nm with the common resolution of 8 nm.

The NIR lights are further delivered to the pomelo samples through a pushbroom scanner. The scanner includes a flat mirror and a transflective mirror as its main optical parts. The pushbroom scanner uses a horizontally movable back-and-forth motion to form the spatial dimensions of the hyperspectral image. It is steadily set 20 cm away from the surface plane of the sample pool in the vertical direction. The reflectance lights that come out from the sample enter an MT-CT image detector, which includes a CCD unit and some necessary fundamental optical parts. The NIRHI spectral data are finally recorded at the data output segment, where there is always a high-performance computer.

### Sample Preparation and Data Acquisition

A total of 300 mature pomelo fruits were collected from a pomelo forest in southern China. An elementary pre-experimental selection was made before measuring contents. Some fruits with a homogeneous peel surface were reserved for further detection. Some fruits with flesh that had minimal moisture were removed from the experiments. A total of 248 pomelo fruits were selected as the target samples for NIRHI measurements and conventional chemical detection.

Each of the 248 target fruits was cut into two halves along its central longitudinal surface. One half of each fruit was sent to quantify its contents of SU, VC, and OA contents. These three analytes should be detected on the cutting interface via different chemical experiments. These chemically measured contents were supposed to be the reference values for NIRHI modeling because the analyte fruit flesh samples were from the same cutting surface. The SU content was identified by 3,5-dinitrosalicylic acid colorimetry (China's agricultural industry standard, NY/T 2742-2015). The VC content was determined by 2,6-dichloro-indophenol titration (China's national standard, GB 5009.86-2016). The OA content was detected by ion chromatography (China's national standard, GB 5009.157-2016). The descriptive statistics for the SU, VC, and OA contents of the 248 pomelo fruit samples are shown in Table 1.

**Table 1:** The descriptive statistics for the SU, VC, and OA of the 248 pomelo fruit samples.

	**Maximum**	**Minimum**	**Average**	**Standard deviation**
Sugar (SU, %)	14.52	8.63	11.51	1.78
Vitamin C (VC, mg/kg)	623.02	347.51	487.71	82.91
Organic acid (OA, g/kg)	13.68	8.91	11.12	1.39

**Figure 2:**
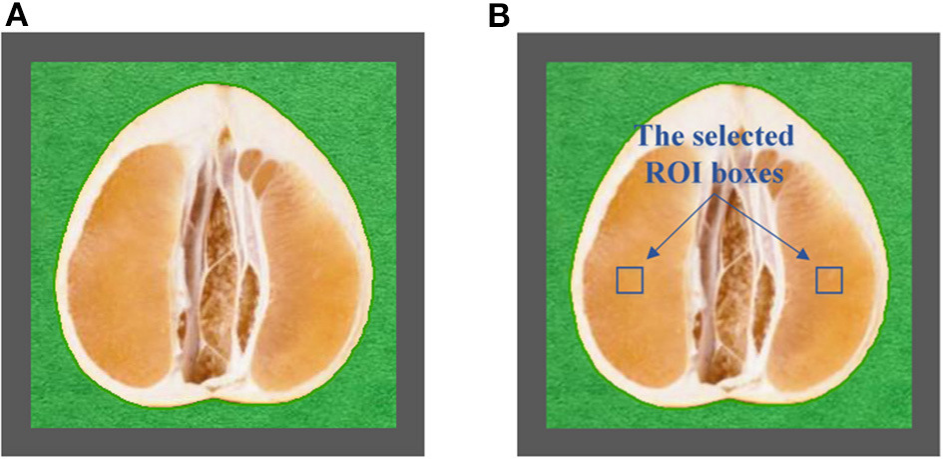
The central longitudinal cut view of the equipped pomelo sample **(A)** and the selected ROI areas **(B)**.

**Figure 3:**
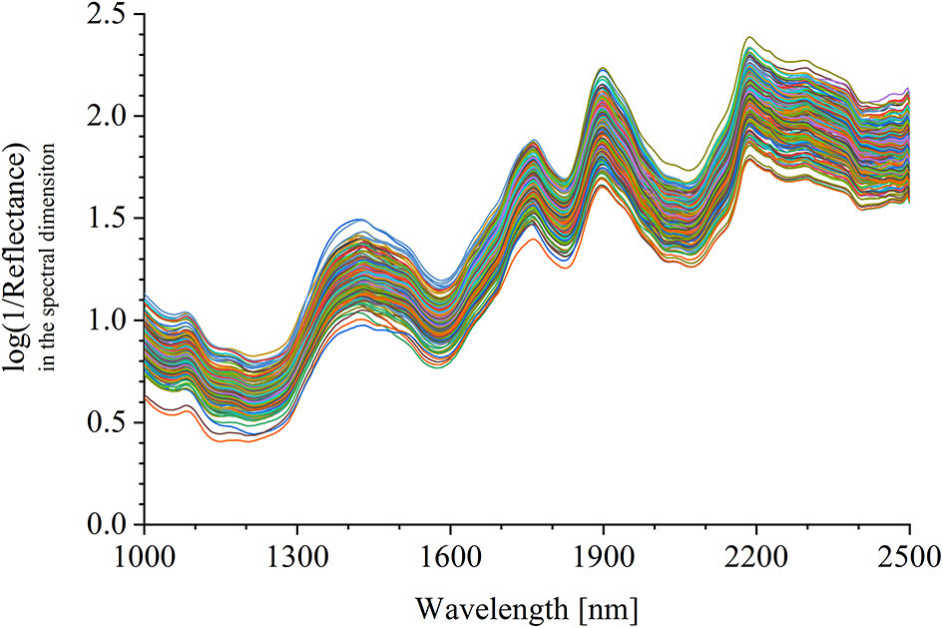
The extracted average spectra of the 248 pomelo samples in the NIRHI spectral dimension.

The half was equipped in the sample pool accessory. As shown in Figure 2A, the halved pomelo fruit was placed into the cuboid sample pool (the gray frame). The interspace between the fruit and the box was filled with plasticine (the green part). The filled sample pool was equipped to the constructed hyperspectral system, and the NIRHI spectral data of this sample were collected by pushbroom scanning. The NIRHI data have two spatial dimensions and one spectral dimension. In the spatial dimensions, the selection of ROI was studied in our previous work (Chen et al., 2019), which reported that the 5 × 5 square-size data extracted from the core spatial pixel area provide optimal spectroscopy calibration results. Thus, we selected two ROIs of 5 × 5 pixels from the main flesh areas around the fruit-shaped equatorial plane (see the two blue boxes in Figure 2B). The spectral data within these two ROI areas were extracted from the NIRHI spatial-spectral data cube. Fifty pixels of NIR spectra were acquired for each pomelo fruit sample. The average of these 50 spectral data was calculated as the spectral information of each sample for further chemometric modeling. Finally, the average spectra of all 248 pomelo samples were obtained, and the spectral curves are illustrated in Figure 3.

### The RBF-PLS Method

The RBF-implemented PLS method is a provoked kernel PLS regression algorithm extended from the common PLS regression. It uses the RBF kernel function to transform raw non-linear complex data into a new defined feature space, in which the data can be linearly predicted with the tuning of the number of PLS latent variables (Chakraborty, 2012; Goudarzi, 2016). The RBF kernel is defined as follows (Ring and Eskofier, 2016):

$$\begin{array}{l} K\left(x_{i},x_{j}\right)=\exp\left(\frac{-||x_{i}-x_{j}|{|}^{2}}{\sigma^{2}}\right),\;i,j=1,2\ldots n \end{array}$$

where σ represents the kernel width. Different values of σ would lead to diverse kernel mapping results in the new data space. For a fixed value of σ, the function *K*(*x*_*i*_, *x*_*j*_) obtains different computing values for varying training data of *x*_*i*_ and *x*_*j*_, thereby generating the kernel matrix for the *n* training samples, which is constructed as

$$\begin{array}{l} K=\left[\begin{array}{cc} \begin{array}{cc} K\left(x_{1},x_{1}\right) & K\left(x_{1},x_{2}\right)\\ K\left(x_{2},x_{1}\right) & K\left(x_{2},x_{2}\right) \end{array} & \begin{array}{cc} \cdots & K\left(x_{1},x_{n}\right)\\ \cdots & K\left(x_{2},x_{n}\right) \end{array}\\ \begin{array}{cc} \vdots \; & \vdots \\ K\left(x_{n},x_{1}\right) & K\left(x_{n},x_{2}\right) \end{array} & \begin{array}{cc} \ddots & \vdots \;\\ \cdots & K\left(x_{n},x_{n}\right) \end{array}\\ \; \end{array}\right], \end{array}$$

Successively, the matrix *K* is transformed to *M*,

$$\begin{array}{l} M=K-\frac{1}{n}I_{n}K-\frac{1}{n}KI_{n}+\;\frac{1}{n^{2}}I_{n}KI_{n}, \end{array}$$

where *I*_*n*_ is an *n*-dimensional square all-one matrix. To make the method smart and data-driven, the algorithm of RBF-PLS training can be operated in an iteration process as follows:

**Table:** 

Step 1: *E* = *M, F* = *Y*;
Step 2: Randomly initialize *U* (a matrix consists of *s* latent variables);
Step 3: *V* = *KU*, *V* ← *V*/||*V*||;
Step 4: *C* = *Y*^T^*V*;
Step 5: *U* = *YC*, *U* ← *U*/||*U*||;
Step 6: Repeat Steps 3–6 until convergence occurs;
Step 7: Residual matrix *E* and *F* were computed, *E* ← (*I* − *VV*^*T*^)*E*(*I* − *VV*^*T*^), *F* ← *F* − *VV*^*T*^*Y*, where *I* is an *n*-dimensional identity matrix;
Step 8: Turn to Step 3 until the convergence occurs for the residuals *E* and *F*.

The predicted data of training set are evaluated by the equation

$$\begin{array}{l} Y^{\prime }=MU{\left(V^{T}MU\right)}^{-\;1}V^{T}Y, \end{array}$$

where *V* is formed by the columns of latent vector *v*; *U* is formed by the columns of latent vector *u*; and *Y* is the predictor matrix. The training process shows that the optimization of the RBF-PLS calibration model is mainly controlled by tuning the RBF kernel width (i.e., σ) and the number of PLS latent variables (i.e., *s*). The combined optimization of σ and *s* should be an applicable machine learning mode for advanced parameter training.

Furthermore, for the testing sample set, the kernel matrix *K*_test_ is computed and constructed similar to constructing *K*, and *K*_test_ is (*t* × *n*)-dimensional. Each element of *K*_test_ is obtained by computing the kernel function between the *t* testing samples and the *n* training samples. Successively, we will have *K*_test_ transformed to *M*_test_:

$$\begin{array}{l} M_{\text{test}}=K_{\text{test}}-\frac{1}{n}I_{t}K-\frac{1}{n}K_{\text{test}}I_{n}+\;\frac{1}{n^{2}}I_{t}KI_{n}, \end{array}$$

where *I*_*t*_ is a *t* × *n* all-one matrix. The algorithm of the testing part is similar to that of the training part, and the prediction equation of the testing set has the same structure as that of the training set.

The RBF-PLS model is developed by regression of the response matrix *X* against the predictor matrix *Y*. The model based on experimental data is established to quantitatively estimate the pseudo-unknown samples based on their measured features. RBF-PLS regression and prediction were carried out using the MATLAB coding platform (ver. R2018a) accompanied with its toolboxes. The parametric scaling on the kernel function can be launched in a deep learning mode, and the selection of latent variables can be embedded for deep combined optimization.

### Model Evaluation Indicators

The chemometric study for the NIRHI analytical model requires the method to be intelligently adjusted to the detected data. Thus, the data knowledge should be recognized in a self-adaptive machine learning mode. On this basis, the NIRHI spectral data of all 248 pomelo fruit samples should be primarily divided into the training set and the testing set. The training samples are used to establish and optimize the model. By contrast, the testing samples, which are not involved in the model training process, aim to evaluate the best-trained calibration model. The model optimization effects need to be validated during the training process, so the training sample set should be further divided into two subsets: the calibration set and the validation set. The calibration set is for model establishment, and the validation set is for model optimization.

Experimental evidence showed that the samples divided for calibration, validation, and testing are usually in the ratio of 2:1:1 (Chen et al., 2015). Of the total 248 samples, we randomly chose 120 samples for calibration, 64 samples for validation, and 64 samples for testing.

The model prediction effects are generally quantified using two important indicators. One is the root mean square error (RMSE), which is used to estimate the model prediction bias. The other one is the correlation coefficient (CC), which is a statistical metric representing the closeness of the NIRHI predicted values to the chemically measured reference contents. These two indicators are formulated as follows:

$$\begin{array}{l} \text{RMSE}=\sqrt{\frac{1}{n-1}\overset{n}{\underset{i=1}{\sum }}{\left(y_{i}-{ŷ}_{i}\right)}^{2}},\\ \text{ CC}=\frac{\sum_{i=1}^{n}\left(y_{i}-y_{\text{ave}}\right)\left({ŷ}_{i}-{ŷ}_{\text{ave}}\right)}{\sqrt{\sum {\left(y_{i}-y_{\text{ave}}\right)}^{2}\sum {\left({ŷ}_{i}-\;{ŷ}_{\text{ave}}\right)}^{2}}}, \end{array}$$

where ŷ_*i*_ and *y*_*i*_ are the NIRHI predicted value and its chemically measured reference value of the *i*-th sample, respectively. ŷ_ave_ and *y*_ave_ are the average predicted value and average reference value of *n* samples, respectively. *n* is the total number of target samples.

As the best optimal model was identified by the validation samples and evaluated by the testing samples, the model indicators were denoted as RMSE_V_ and CC_V_ for the validation sample set and denoted as RMSE_T_ and CC_T_ for the testing sample set.

## Results and Discussions

### Parametric Scaling Deep Learning Results of the RBF-PLS Model

**Figure 4:**
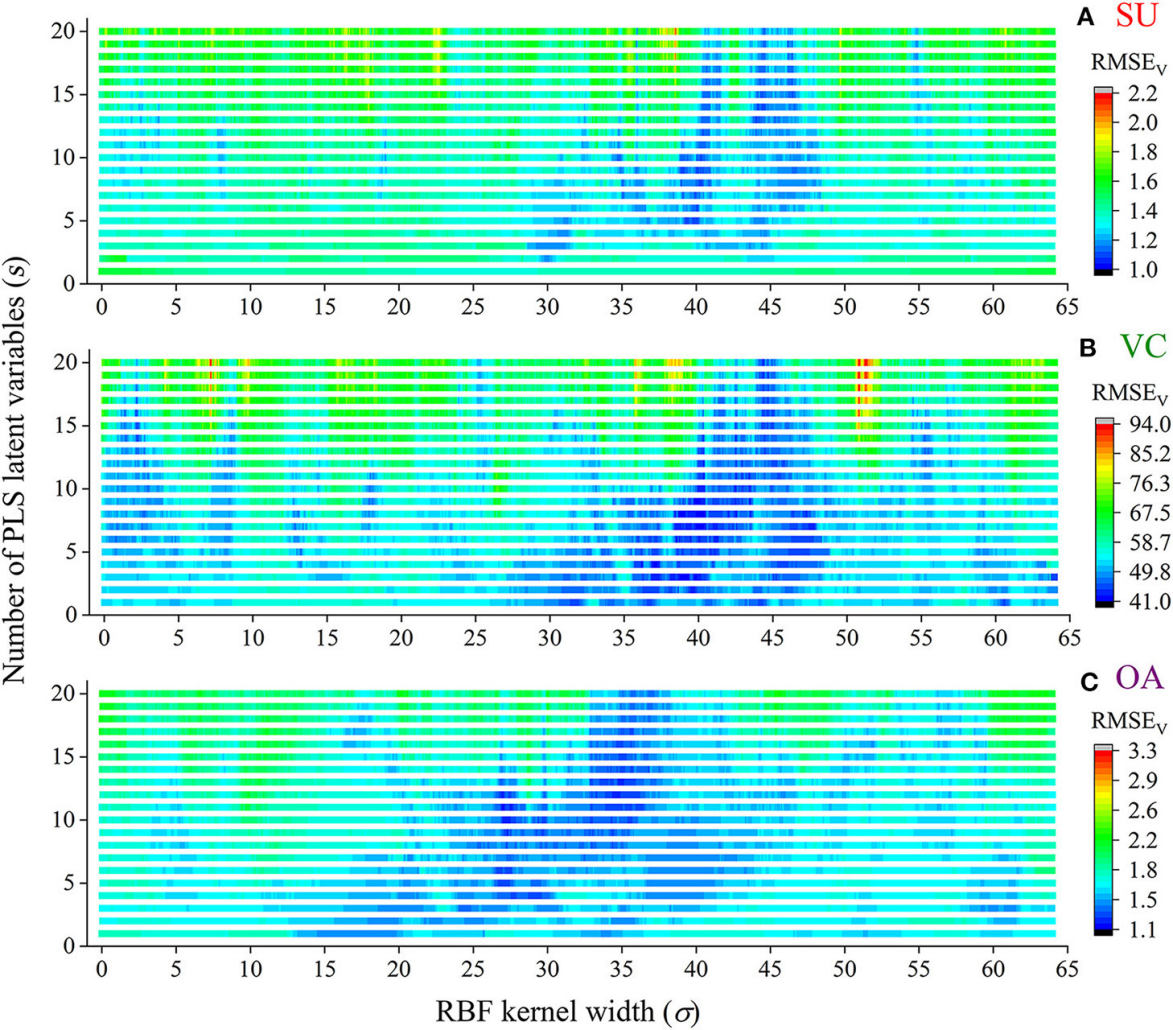
The combined deep tuning of RBF kernel width (σ) and the number of PLS latent variables (*s*) for the optimization of the NIRHI calibration model (**A–C** are for the prediction of SU, VC, and OA, respectively).

The extracted NIRHI spectra of the 248 pomelo fruit samples were used to establish calibration models by using the proposed RBF-PLS method. A deep learning architecture was built for parametric scaling search of the optimal RBF-PLS parameters. The models for predicting the SU, VC, and OA contents were trained based on the 128 calibration samples, and the modeling parameters were tunable for deep searching of their optimal combination values. For the RBF kernel, the kernel width (σ) is commonly set as 2^*i*^ (*i* = 0, ±1, ±2…) (Menezes et al., 2019). For statistical convenience, we set σ to change from 0.01 to 64 with the step of 0.01, which included the close estimation of 2^*i*^ with *i* = 0, ±1, ±2, ±3, ±4, ±5, ±6. Thus, there were 6,400 candidate values of σ for the kernel width scaling. Meanwhile, the PLS latent variable queues in the front were considered the most informative for spectral data interpretation (Shariati-Rad and Hasani, 2010). The number of latent variables (*s*) was set as integers from 1 to 20, which indicated that the most important latent variables were used for model optimization. The predictive RMSE_V_ of the validation samples was used as the main indicator to identify the appreciating model with its optimal parameter combination of (σ, *s*). The grid search of the RMSE_V_ corresponding to each combination is shown in Figure 4. In Figure 4, the two-dimensional axes represent the parametric tuning of σ and *s*, respectively. The predictive RMSE_V_ values of each model were demonstrated as contour color mappings. Figures 4A–C show the validation results for the SU, VC, and OA contents, respectively. The most optimal training results could be found at the dark blue digit locations, so the optimal combination of (σ, *s*) was identified (see Table 2). The corresponding modeling results (RMSE_V_ and CC_V_) were also listed in the table. For comparison, the classical PLS model was established in model training, and the results are listed in Table 2. The prediction results in Table 2 indicated that the RBF-PLS models performed better than the PLS model during the training process. Therefore, the RBF-PLS models are feasible for the NIRHI quantitative determination of the designated contents related to the quality of pomelo fruits.

**Table 2:** The optimal RBF-PLS models for NIRHI prediction of SU, VC, and OA contents in pomelo fruit samples.

	**RBR-PLS model**			**PLS model**	
	**Kernel parameters**	**RMSE_**V**_**	**CC_**V**_**	**RMSE_**V**_**	**CC_**V**_**
SU (%)	σ = 38.97; *s* = 8	1.076%	0.921	1.361%	0.895
VC (mg/kg)	σ = 44.38; *s* = 14	41.381 mg/kg	0.913	50.672 mg/kg	0.862
OA (g/kg)	σ = 27.20; *s* = 11	1.136 g/kg	0.902	1.475 g/kg	0.875

### Iteration Progress for the Selection of PLS Latent Variables

**Figure 5:**
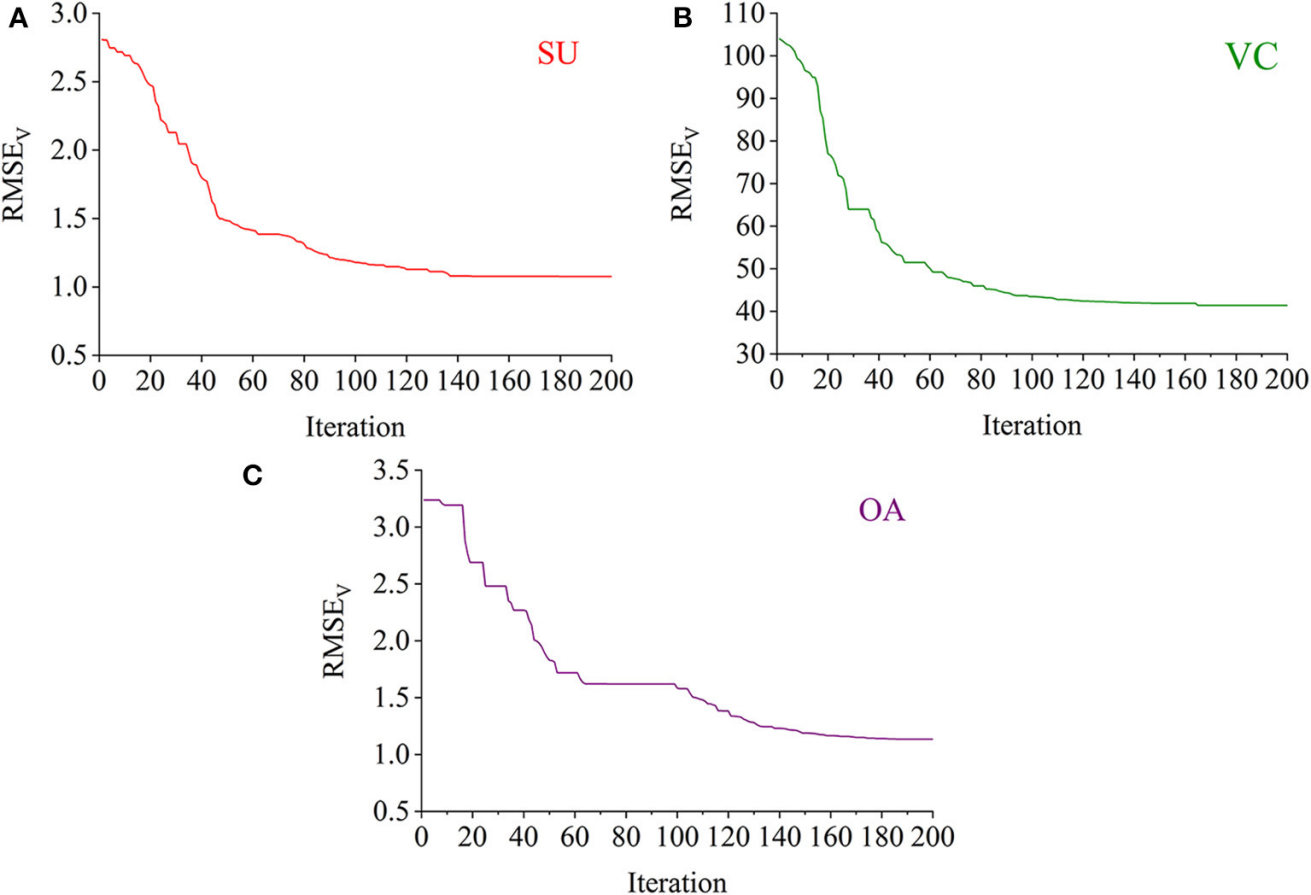
The iterative optimization of the RBF-PLS models for the NIRHI data training (**A–C** are for the prediction of SU, VC, and OA, respectively).

The RBF-PLS model was optimized by iterative updating of the matrix of latent variables (i.e., the matrix *U*). For a fixed value of *s*, the applied latent variables were randomly initialized and then gradually alternated. The progress of updating the latent variables was iteratively set for 200 times. For example, the optimal model for the prediction of the SU content was determined with eight latent variables. The eight latent variables were randomly chosen at the beginning, and they gave the initial predictive RMSE_V_ of 2.807%. The 200-time iteration model made the prediction more accurate as the RMSE_V_ curve went down and became stable (see Figure 5A). Finally, the prediction on the SU content with eight latent variables observed its optimal result of RMSE_V_ equal to 1.076%, which was captured within the 200 iteration time. Similarly, the iterative optimization trends of the RBF-PLS models for the prediction of the VC and OA contents are shown in Figures 5B,C. As shown in Figure 5, the iteration mechanism during the PLS process is feasible to enhance the optimization ability of the RBF-PLS calibration model for the NIRHI spectral analysis of pomelo fruit samples.

### Model Evaluation Based on the Testing Samples

**Figure 6:**
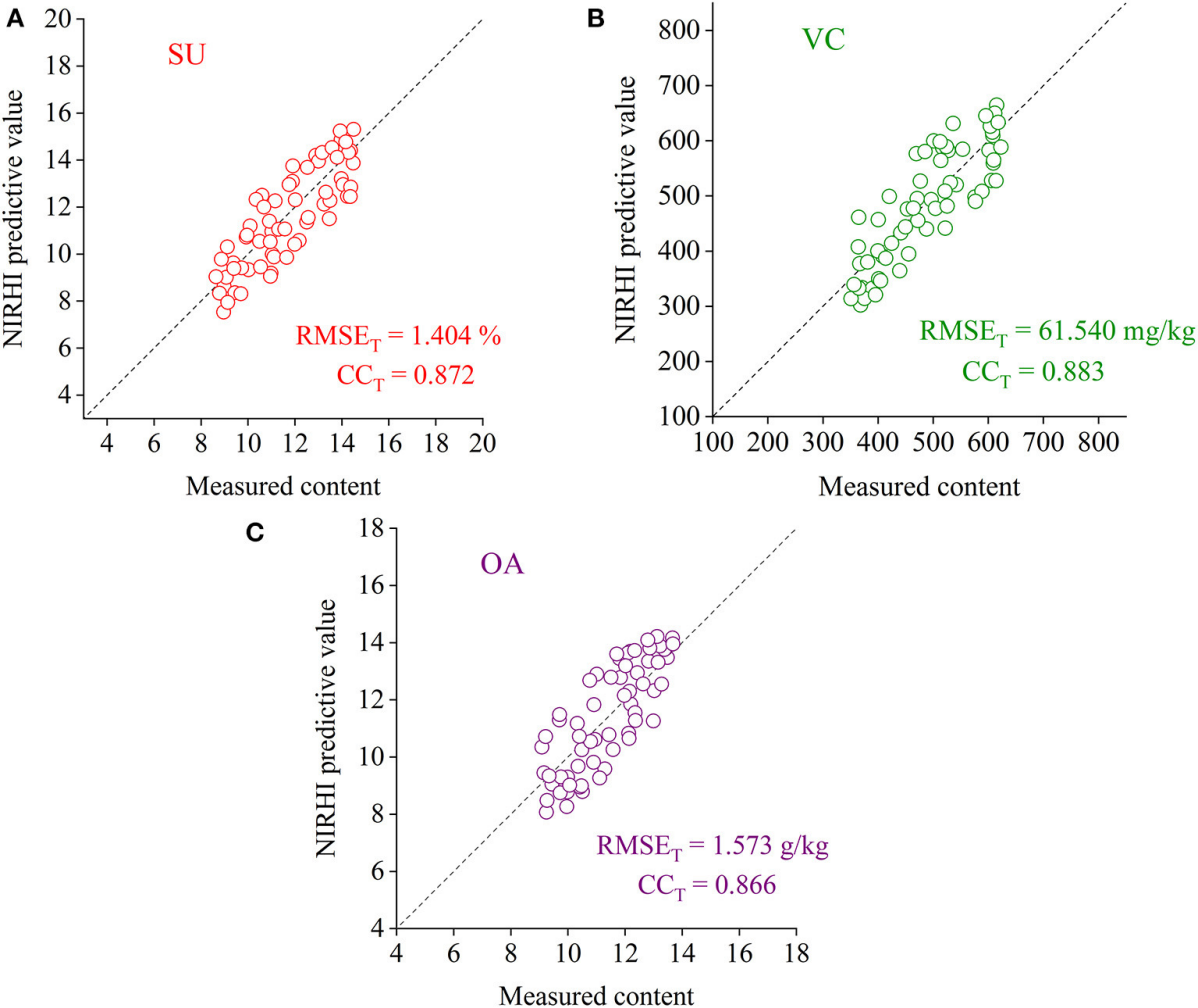
The regression plots of the NIRHI predictions and the reference chemical measurements for the testing samples (**A–C** are for the prediction of SU, VC, and OA, respectively).

To verify the effectiveness of the RBF-PLS model applied to the NIRHI spectral analysis of pomelo fruit samples, the well-trained models for the prediction of the SU, VC, and OA contents were evaluated by the testing samples, which were not involved in the modeling process. The testing models were re-established by using the optimally selected parameter combination of (σ, *s*), as shown in Table 2. The regression plots of the NIRHI predictions and the reference chemical measurements are shown in Figure 6. The predicted RMSE_T_ values were obtained as 1.404%, 61.540 mg/kg, and 1.573 g/kg for the model testing on the SU, VC, and OA contents, respectively, which were under 15% of their reference chemical measurements. The acquired CC_T_ was larger than 0.85, which seemed to be acceptable for model evaluation of agricultural products.

## Conclusions

The RBF-PLS method was proposed to extract the spectral features from the NIRHI data for the quantitative determination of the SU, VC, and OA contents in pomelo samples. The NIRHI spatial properties were pre-determined based on previous research results. The spectral calibration models were trained in the deep search of the combined parameters (σ, *s*), where σ was screened from 6,400 possible candidate values changing from 0.01 to 64 with a step of 0.01, and *s* was changed as an integer from 1 to 20. To observe the minimum RMSE_V_ and CC_V_, the grid values of (σ, *s*) were all tested, and the optimal parameters were identified. The optimal models were found during the calibration and validation processes, with the predictive results of RMSE_V_ equal to 1.076% for SU, 41.381 mg/kg for VC, and 1.136 g/kg for OA. All of the three CC_V_ exceeded 0.9. The selected models were evaluated based on the testing samples, and the prediction results were also appreciable. The experimental results indicated that the proposed parametric scaling RBF-PLS method is feasible to determine some pomelo fruit quality targeting contents in combination with the NIRHI technology. Studies on NIRHI chemometric methods are essential to improve the calibration models in the rapid determination of agricultural products.

## Data Availability Statement

The raw data supporting the conclusions of this article will be made available by the authors, without undue reservation.

## Author Contributions

HC: conceptualization, methodology, writing–original draft, and writing–review & editing. HQ: data curation and formal analysis. QF: investigation and validation. LX: writing–original draft. QL: software and visualization. KC: resources, supervision, and writing–review & editing. All authors contributed to the article and approved the submitted version.

## Conflict of Interest

The authors declare that the research was conducted in the absence of any commercial or financial relationships that could be construed as a potential conflict of interest.
